# Glucose control in diabetes during home confinement for the first pandemic wave of COVID-19: a meta-analysis of observational studies

**DOI:** 10.1007/s00592-021-01754-2

**Published:** 2021-06-22

**Authors:** Giovanni Antonio Silverii, Chiara Delli Poggi, Ilaria Dicembrini, Matteo Monami, Edoardo Mannucci

**Affiliations:** Diabetes Unit, Experimental and Clinical Biomedical Sciences “Mario Serio” Department, AOU Careggi Hospital, University of Florence, Largo Brambilla 3, 50134 Florence, Italy

**Keywords:** COVID-19, Diabetes mellitus, Lockdown, Meta analysis

## Abstract

**Aim:**

To assess the effect on glycaemic control of confinement due to lockdown measures, during COVID-19 pandemic, in people with type 1 (T1DM) and type 2 (T2DM) diabetes.

**Methods:**

Meta-analysis of observational studies reporting measures of glucose control and variability before and during and/or after periods of confinement caused by COVID-19 in 2020 and/or 2021.

**Results:**

We included 27 studies on T1DM. No significant change in Hba1c was observed after lockdown (WMD − 1.474 [− 3.26; 0.31] mmol/mol, *I*^2^ = 93.9). TIR significantly increased during and after lockdown (WMD: 2.73 1.47; 4.23 %, *I*^2^ = 81% and 3.73 [1.13; 5.33] %, *I*^2^ = 85%, respectively).We retrieved nine studies on T2DM patients. No significant variation in HbA1c was detected (WMD − 1.257 − 3.91; 1.39 mmol/mol, *I*^2^ = 98.3%). HbA1c had a more favourable trend in studies performed in Asia than in Europe (*p* = 0.022 between groups).

**Conclusion:**

Lockdown showed no significant detrimental effect on HbA1c in either T1DM or T2DM. Conversely, home confinement led to a reduction in mean glucose and glucose variability in T1DM, although with a high heterogeneity of results.

**Supplementary Information:**

The online version contains supplementary material available at 10.1007/s00592-021-01754-2.

## Introduction

COVID-19 pandemic forced most Countries to adopt confinement measures to prevent the spreading of the disease. Those measures, although different across countries, all led to some extent to a reduction in physical activity, with the shutdown of gyms in many countries; moreover, the commitment to stay at home modified daily routine, increasing the time available for cooking and eating. All those changes in daily routine may have altered glycaemic control in people with diabetes mellitus [1].

The outbreak of COVID-19 during the spring of 2020 and the consequent lockdown in many countries also reduced the access to diabetes specialist care [2], metabolic monitoring through laboratory determinations [3], and visits and examinations for screening of diabetic complications. The reduced availability of medical care, associated with insufficient patient self-management [4], was a possible determinant of the observed increase in incidence and severity of diabetic complications, such as foot ulcers [5]. In order to maintain basic care, telemonitoring was implemented in many countries [6–8]. Telematic interactions were also used to help patients in developing coping strategies for managing home confinement [9–11] and maintaining physical activity [12, 13]. Physical activity is crucial in diabetes mellitus management, especially during a pandemic, as it enhances immune response to viral infections [14]; therefore, many efforts have been performed to help patients in finding strategies to maintain it during lockdown [15].

The success of strategies implemented for the care of diabetes during prolonged lockdown for COVID-19 epidemic waves was assessed in several observational studies, providing discordant results [16–18]. This meta-analysis is aimed at collecting all evidence on the effect on glycaemic control of confinement due to lockdown measures, and the consequent adaptation of care, during the first wave of COVID-19 pandemic, in patients with type 1 and type 2 diabetes.

## Materials and methods

Review Protocol has been submitted for registration to the PROSPERO website CRD42021234360https://www.crd.york.ac.uk/PROSPERO/. Searches were performed in PubMed and Embase (“COVID-19” and “diabetes mellitus”) up to March 10, 2021 (see the complete search strings in Supplementary Table 1). Further studies were searched among references from papers.

Observational studies written in English language and performed on humans, enrolling patients with type 1 or type 2 diabetes, and reporting measures of glucose control and variability before and during and/or after periods of confinement caused by COVID-19 in 2021 and/or 2021 were included.

The principal endpoints were variations from baseline in glycated haemoglobin (HbA1c) and Time in Range [19] (TIR, time during which glycaemia is maintained between 70 and 180 mg/dl) during and after lockdown. Additional outcomes were Time Above Range (TAR, time during which glycaemia is above 180 mg/dl), Time Below Range (TBR, time during which glycaemia is below 70 mg/dl), mean glucose, glucose variability (coefficient of variation), frequency of glucose monitoring, variations in eating habits and physical activity, perceived stress.

The following data were extracted: number of included patients, duration of diabetes, mean age, proportion of male patients, patients using flash glucose monitoring (FGM), continuous glucose monitoring (CGM), or self-monitoring of blood glucose (SMBG); proportion of patients in continuous subcutaneous insulin infusion (CSII) multiple daily insulin injections (MDI), basal insulin only, sodium glucose transporter 2 inhibitors (SGLT2-i), dipeptiydil-4 inhibitors (DPP-4i), pioglitazone, metformin, sulphonylureas (SU); study duration, country of origin, duration of Lockdown, use of teleconsulting, values of HbA1c before and after lockdown, TIR,TAR, TBR), Coefficient of variability (CV), use of telemedicine, any variation in physical activity, diet, stress. The quality of the studies was assessed at study level, using the scale developed by Carmen-Moga and colleagues (Table 2S).

Titles and abstracts were screened independently by two authors. If one or more inclusion criteria were present, the whole article was read, in order to assess if all the inclusion criteria were present. Study selection, data retrieval and study quality assessment were performed independently by two investigators (C.D.P. and G.A.S.) and conflicts resolved by a third investigator (M.M.).

Begg’s and Mandzumkar test were used to detect publication bias, with reference to all principal endpoints; funnel plots were used when more than 10 studies were available for the specific outcome. Weighed mean differences (WMD) during and after lockdown vs. pre-lockdown), with 95% confidence intervals, were calculated using random effect models. Rosenthal's conservative estimate of 0.7 [20] was adopted for pre-post correlation. Fixed effect models were used for sensitivity analysis. *I*^2^ statistics was used for the assessment of heterogeneity. Subgroup analyses were performed, based on country, age group (children and adolescent < 18 years, adult > 18 years), type of monitoring (FGM, CGM, SMBG), insulin treatment (multiple injections, continuous subcutaneous infusion, hybrid closed-loop systems), structured telemonitoring (yes/no).

All the analyses were performed on Comprehensive Meta-Analysis Software, V3 edition, Biostat Inc. 14 North Dean Street, Englewood, NJ 07,631, USA.

## Results

**Fig. 1:**
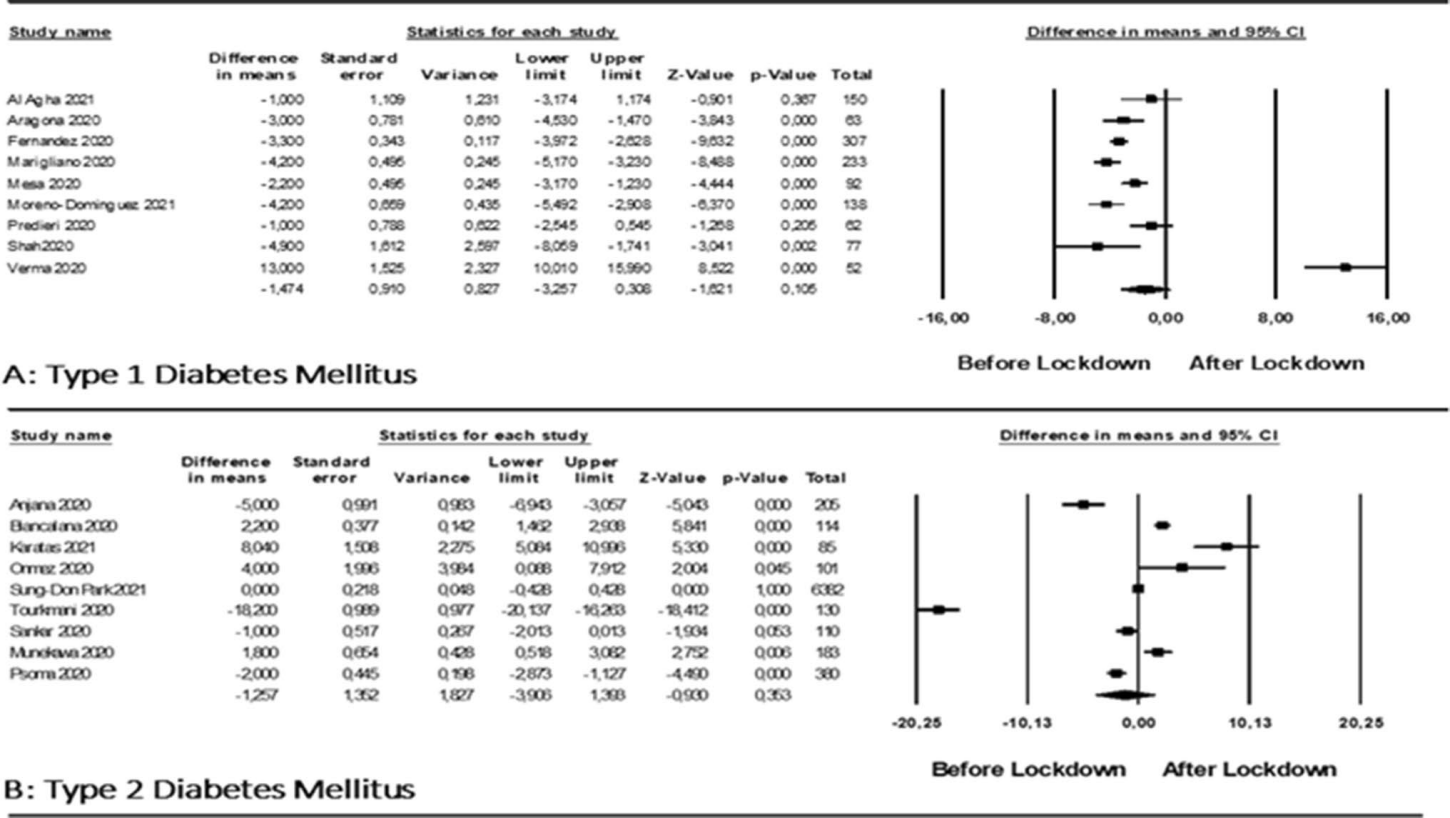
Difference in HBA1c (mmol/mol), before and after lockdown, in people with T1DM (panel A) and in people with T2DM (panel B)

Out of 1634 results, 122 studies were selected on the basis of the titles and abstracts. Of those, 79 did not report data on glycaemic control during or after lockdown measures; 6 included both T1DM and T2DM patients without providing subgroup analysis [17]; two studies reported subgroup analyses with no overall analysis [21]. Thirty-six studies reported glycaemic control before and during or after the pandemic restrictions and were therefore included in the meta-analysis (Fig. 1S). Of those, 9 were performed in type 2 diabetes, whereas 27 were performed in type 1 diabetes. Characteristics of the included studies are reported in Table 1.

**Table 1:** Characteristics of the included studies

Study	DM	FGM	CGM	SMBG	CSII	MDI	LAI	Met	DPP4i	SGLT2i	GLP1RA	PIO	SU	Country	N	Age	M	DM dur	Tele
Al Agha 2021 [32]	1	0	39	61	9.3	91	0	0	0	0	0	0	0	Arabia	150	13	32	4.2	0
Aragona 2020 [33]	1	82	18	0	44	56	0	4	0	0	0	0	0	Italy	63	44	44	22	0
Barchetta 2020 [34]	1	56	44	0	44	56	0	0	0	0	0	0	0	Italy	50	41	62	18	1
Barmpagianni 2021 [35]	1	22	88	0	100	0	0	0	0	0	0	0	0	Greece	46	38	35	19.5	0
Brener 2020 [36]	1	0	100	0	73	27	0	0	0	0	0	0	0	Israel	102	11	51	4.2	0
Capaldo 2020 [37]	1	63	37	0	50	50	0	0	0	0	0	0	0	Italy	207	38	54	NR	1
Caruso 2020 [38]	1	100	0	0	19	81	0	0	0	0	0	0	0	Italy	48	42	52	NR	1
Ceconi 2020 [39]	1	0	100	0	100	0	0	0	0	0	0	0	0	Italy	13	14	62	8.1	0
Di Dalmazi 2020 [40]	1	67	33	0	34	66	0	0	0	0	0	0	0	Italy	130	23	55	11.1	1
Dover 2021 [41]	1	100	0	0	26	74	0	0	0	0	0	0	0	UK	572	39	53	18	NR
Fernandez 2020 [42]	1	100	0	0	7	94	0	0	0	0	0	0	0	Spain	307	46	50	21.1	1
Marigliano 2020 [43]	1	42	43	15	38	62	0	0	0	0	0	0	0	Italy	233	14	56	7	NR
Mesa 2020 [44]	1	81	19	0	0	100	0	0	0	0	0	0	0	Spain	92	43	57	23.1	1
Moreno-Dominguez 2021 [45]	1	100	0	0	19	81	0	0	0	0	0	0	0	Spain	138	43	36	21.7	NR
Pla 2020 [46]	1	100	0	0	10	90	0	0	0	0	0	0	0	Spain	50	43	46	24.1	1
Predieri 2020 [47]	1	0	100	0	47	53	0	0	0	0	0	0	0	Italy	62	11	50	4.9	1
Shah2020 [48]	1	0	0	100	4	96	0	0	0	0	0	0	0	India	77		58	5.9	1
Verma 2020 [49]	1	0	0	100	0	100	0	0	0	0	0	0	0	India	52	12	42	NR	1
Vinals 2020 [50]	1	0	100	0	100	0	0	0	0	0	0	0	0	Spain	59	46	44	30	NR
Cotovad-Bellas 2021 [51]	1	100	0	0	100	0	0	0	0	0	0	0	0	Spain	44	37	33	NR	1
Prabhu Navis 2020 [52]	1	71	29	0	70	30	0	0	0	0	0	0	0	Uk	223	41	54	23.6	1
Christoforidis 2020 [53]	1	0	100	0	100	0	0	0	0	0	0	0	0	Greece	34	11	47	5.14	NR
Dovc 2020 [54]	1	NR	36	NR	48	52	0	0	0	0	0	0	0	Slovenia	326	14	45	6.3	1
Longo 2020 [55]	1	NR	100	NR	100	0	0	0	0	0	0	0	0	Italy	30	32	43	9.5	1
Schiaffini 2020 [56]	1	0	100	0	100	0	0	0	0	0	0	0	0	Italy	22	9	63	NR	0
Odeh 2020 [57]	1	NR	NR	NR	NR	NR	0	0	0	0	0	0	0	Jordan	97	11	49	NR	0
Assaloni 2020 [58]	1	NR	NR	NR	NR	NR	0	0	0	0	0	0	0	Italy	154	45	55	NR	0
Anjana 2020 [23]	2	NR	NR	NR	NR									India	2510	54	52	14.3	1
Biancalana 2020 [59]	2	NR	NR	NR	0	19.3		76.3						Italy	114	69	62	8.4	1
Karatas 2020 [60]	2	NR	NR	NR	0	62.2		89.3						Turkey	85	55	32	11.7	NR
Onmez 2020 [24]	2	NR	NR	NR	0	16		69	54	22	0	12	15	Turkey	101	55	56	7.5	NR
Sung-Don Park 2021 [30]	2	NR	NR	NR	NR									S. Korea	6382	63	57	NR	NR
Tourkmani 2020 [61]	2	NR	NR	NR	0	57.6	8.5	84.6	46.5	NR	11.5	1.5	30	Saudi Ar	130	57	0	14	1
Sankar 2020 [22]	2	NR	NR	NR	0	46.4		100						India	110	59	47	NR	1
Munekawa 2020 [25]	2	NR	NR	NR	0	33.5		83.7						Japan	159	67.4	0	14.4	1

*DM* diabetes mellitus, *FGM* flash glucose monitoring, *CGM* continuous glucose monitoring, *SMBG* self-monitoring blood glucose, *CSII* continuous subcutaneous insulin infusion, *MDI* multiple daily insulin injections, *LAI* long acting insulin, *Met* metformin, *DPP4i* dipeptiydil-4 inhibitors, *SGLT2i* sodium glucose transporter 2 inhibitors, *GLP1RA* glucagon-like peptide receptor agonist, *PIO* pioglitazone, *SU* sulphonylureas, *N* number, *M* males, Diabetes Mellitus, *dur* duration, *NR* not reported

### Type 1 diabetes

*HbA1c.* Only 9 studies, enrolling 1174 patients, reported HbA1c before and after lockdown in patients with type 1 diabetes. Mean age was 31.3 years. No significant change in Hba1c was observed after lockdown (WMD − 1.474 [− 3.26; 0.31] mmol/mol; Fig. 1 panel A), with relevant heterogeneity (*I*^2^ = 93.9). No publication bias was detected (Kendall’s tau: 16 *p* = 0.1). A subgroup analysis showed that studies with more than 50% of enrolled patients on continuous glucose monitoring showed a significant decrease in HbA1c (WMD − 3.00 [− 4.84; − 1.16] mmol/mol), whereas those enrolling more than 50% patients on SMBG showed no significant variation in HbA1c (*p* = 0.003 for difference between groups; Fig. 5S). Studies performed in Europe showed a significant reduction in HbA1c (WMD − 3.053 [ − 3.9; − 2.2] mmol/mol), whereas those performed in Asia did not (WMD; 2.36 [ − 7.50; 12.25] mmol/mol; *p* < 0.0001 for difference between groups). No significant difference was found in subgroup analyses based on age (Table 4S).

#### Time in range

**Fig. 2:**
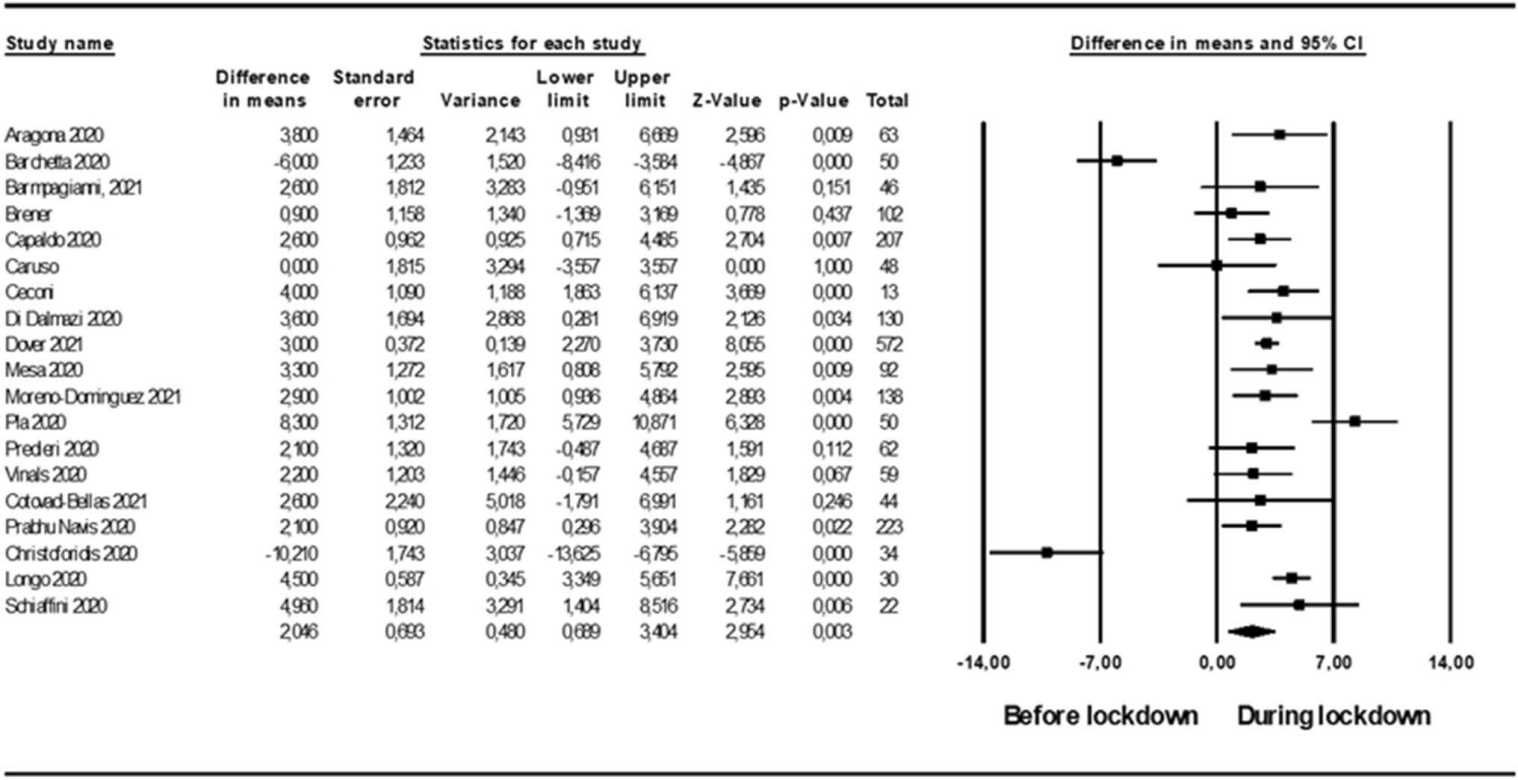
Difference in time in range (%), before and during lockdown, in people with T1DM

Nineteen studies, enrolling 1985 patients, and 10 studies, enrolling 1123 patients, reported information on TIR during and after lockdown, respectively. TIR significantly increased during lockdown (WMD: 2.73 [1.47; 4.23]%; Fig. 2), with high heterogeneity (*I*^2^ = 81%) and no detectable publication bias (Kendall’s tau: 0.1, *p* = 0.59; Fig. 2S, for funnel plot). Subgroups analyses revealed no difference between studies performed in different countries or in different age groups (Fig. 7S). Meta-regression analysis showed no correlation of TIR variation with its baseline value, or with the proportion of subjects on CSII or MDI (Tab. 4S). Conversely, an inverse correlation was detected between variation in TIR and proportion of men among enrolled subjects (Tab 4S, Fig. 3S). TIR was significantly higher after lockdown (WMD 3.73 [1.13; 5.33] %; Fig. 8S) with high heterogeneity (*I*^2^ = 85%). No significant publication bias was detected (Kendall’s tau: − 7. *p* = 0.48).

#### Time above range

TAR during lockdown and after lockdown was reported in 14 and 9 studies, respectively. TAR was significantly lower both during (WMD: − 1.953 [− 2.87; − 1.03] I^2^ = 70, Kendall’s tau: 7. *p* = 0.7; Fig. 9S) and after lockdown (WMD: − 3.49 [ − 0.57; − 1.25] *I*^2^ = 90 Kendall’s tau: 10. *p* = 0.21; Fig. 10S).

#### Time below range

Seventeen studies on type 1 diabetes estimated time below range (TBR) before and during lockdown, whereas nine studies reported TBR before and after lockdown: TBR did not change significantly during (WMD: 0.13 [− 0.18; 0.43]; *I*^2^: 81% and Kendall’s tau: 0.1, *p* = 0.59; Fig. 11S) or after lockdown (WMD: 0.29 [− 0.28; 0.86); *I*^2^ = 94 and Kendall’s tau: 12. *p* = 0.21; Fig. 12S).

#### Mean glucose

In the 14 studies with available data, mean glucose during lockdown was significantly lower than before lockdown (WMD − 2.795 [− 4.816; − 0.774]; Fig. 13S), with high heterogeneity (*I*^2^ = 91) and no evidence of publication bias (Kendall’s tau: − 11.0 *p* = 0.54). In addition, mean glucose was significantly lower after lockdown (WMD − 5.29 [ − 8.055; − 2.53] with high heterogeneity (*I*^2^ = 87.989) and no evidence of publication bias. (Kendall’s tau: − 9 *p* = 0.17; Fig. 14S).

#### Glucose coefficient of variation

Glucose CV during (*n *= 16 studies) and after (*n* = 9 studies) lockdown was significantly reduced (WMD: − 0.97 [− 1.48; − 0.47]; Fig. 15S and − 1.33 [ − 2.11; − 0.56]; Fig. 16S, respectively), with no evidence of publication bias (Kendall’s tau =  − 29.0; *p* = 0.27) and high heterogeneity (*I*^2^ = 79).

#### Patients’ reported behaviours

Thirteen studies enrolling patients with T1DM reported data on patients’ behaviours. The heterogeneity of instruments used for the assessment of patients’ behaviour prevented a formal meta-analysis (Table 2). A reduction in physical activity was reported by 8–70% of patients, whereas those reporting an increase in food intake were 17–46%; moderate-to high stress was found in 20–52% of patients.

**Table 2:** Characteristics of the included studies

Study	Job modifications		Physical activity			Physical activity > 30		Food intake				Stress				
	yes	no	var	%**↑**	%**↓**	before	during	%**↑**	%**↔ **	%**↓**	var	scale	var	high	mod	low
Al Agha 2021	NR	NR	**↓↓**	13	66	68.5	NR	46%	54%	0	NR	NR	NR	NR	NR	NR
Aragona 2020	89	11	NR	NR	NR	NR	35	NR	NR	NR	NR	NR	NR	NR	NR	NR
Assaloni 2020	NR	NR	NR	5	8.5	90.9	82.5	NR	NR	NR	NR	NR	NR	NR	NR	NR
Barchetta 2020	48	52	**↓↓**	NR	NR	52	40	NR	NR	NR	NR	PSS	NR	14	60	26
Capaldo 2020	NR	NR	**↓↓**	19	65	NR	NR	40	NR	NR	**↑↑**	NR	NR	NR	NR	NR
Caruso 2020	48	52	**↓↓**	12	70	NR	NR	42	58	NR	**↑↑**	GHQ‐12	NR	50		50
Di Dalmazi 2020	NR	NR	IPAQ: 1680 METS during lockdown					NR	NR	NR	NR	PSS: 14.5 during lockdown				
Pla 2020	48	52	**↔**	NR	NR	NR	46	NR	NR	NR	NR	NR	**↑**	36	16	48
Predieri 2020	NR	NR	**↓↓↓**	NR	NR	NR	NR	NR	NR	NR	NR	NR	NR	NR	NR	NR
Verma 2020	38	62	**↓**	6	37	NR	NR	17.4	82.6	NR	**↔**	NR	NR	11.5	9.6	78.9
Longo 2020	97	3	NR	NR	NR	NR	20	NR	NR	NR	**↑**	NR	NR	NR	NR	NR
Anjana 2020	NR	NR	**↓**	14	24%	NR	NR	NR	88	NR	**↑**	NR	NR	NR	NR	NR
Onmez 2020	17	NR	**↓↓**	NR	70	NR	NR	55%	45	0	**↑↑**	NR	NR	NR	NR	NR
Sankar 2020	18	NR	**↓**	3	15%	NR	NR	25	12	63	**↓**	NR	**↑**	15	37	52
Munekawa 2020	NR	NR	**↓↓**	NR	51.4	66	NR	20	80	NR	**↑↑**	VAS	**↑**	41.8	NR	NR

Physical activity > 30 = Physical activity for more than 30 min every day, *NR* not reported, *var* variations, job modifications = either remote working or job loss, *mod* moderate, *PSS* perceived stress scale, *IPAQ* international physical activity questionnaire–short form, *MET* metabolic equivalent, *GHQ‐12* general health questionnaire‐12 items arrows, **↓** = slight reduction, **↓↓** = consistent reduction, **↓↓↓** = massive reduction, ↔  = no substantial variation, **↑** = slight increase, **↑↑** = consistent increase

### Type 2 diabetes

Nine studies reporting HbA1c before and after lockdown were available in T2DM patients, including 9591 subjects with a median age of 60.5 years; five studies were performed in Asia (India, South Korea, Japan, Saudi Arabia), whereas four were performed in Europe (Turkey, Italy, Greece). No significant variation in HbA1c was detected (WMD − 1.257 [− 3.91; 1.39] mmol/mol; Fig. 1, panel B), with high heterogeneity (*I*^2^ = 98,3%). No significant publication bias was detected (Kendall’s tau = − 1, *p* = 0.88). A subgroups analysis revealed a significant difference between studies with mean baseline HbA1c below or above 64 mmol/mol (*p* = 0.045 between groups), with those with higher baseline HbA1c showing a greater reduction (Fig. 17S). A further subgroup analysis showed that HbA1c had a more favourable trend in studies performed in Asia than in Europe (*p* = 0.022 between groups) (Fig. 18S). No difference was found between age groups (*p* = 0.22 between studies with a mean age higher or lower than 60 years) (Fig. 19S).

Two studies on people with T2DM [22, 23] both performed in India reported a modest reduction (20–24% of participants) in physical activity with no significant variation in food intake. On the other hand, two studies performed in Turkey and Japan [24, 25] reported a frequent (54–70% of participants) reduction in physical activity and an increase in food intake (20–55% of participants). All the three studies reporting data on stress found a moderate increase in perceived stress and anxiety (Table 2).

## Discussion

Most studies on the glycaemic effects of lockdown were performed in T1DM, usually in patients using either FGM or CGM. The assessment of variations in interstitial glucose can be performed in a shorter time than that required for exploring modifications in HbA1c. For this reason, we already have a substantial body of evidence on the effects of lockdown during the first pandemic wave in T1DM, but not in T2DM.

In T1DM, an improvement in glycaemic control during lockdown was observed, together with a reduction in glucose variability. These results were obtained despite an observed reduction in physical activity and dietary compliance during lockdown, determined by the increased time spent at home; in addition, access to care was impaired during lockdown [26], and surveys on perceived glucose control revealed a high perceived difficulty in dealing with COVID-19 restrictions in people with T1DM [27].

The interpretation of results on glucose control in T1DM is problematic because of their high heterogeneity. The exploration of moderators of lockdown effect, using either subgroup analyses of trials or meta-regression, provides some further insight. Lockdown seems to have produced a greater beneficial effect on females than in males. This is consistent with a Chinese study in which males with T1DM had poorer glycaemic control than females during COVID-19 lockdown [28]; previous findings showed that females with DM, when compared to males, elicited more frequently behaviours aimed at disease prevention, health promotion and symptom recognition [29], which could have been of help in coping with confinement. An increase in glucose monitoring and an improvement in insulin titration during remote working or remote schooling may explain these improvement, as suggested by a study showing an improvement in glucose control only in patients working at home [21]; unfortunately, the information on glucose monitoring and on the proportion of patients on home schooling or working was insufficient to add these variables as moderators. Notably, in studies enrolling a majority of patients with T1DM with interstitial glucose monitoring systems, HbA1c was significantly reduced, suggesting that FGM or CGM could have been a relevant support during confinement. The difference in effects of lockdown between Asian and European studies could have been determined by the different proportion of patients on FGM/CGM (substantially higher in European studies), or to differences in lockdown measures (usually stricter in European countries).

No significant beneficial or detrimental effect of lockdown on glucose control was found in Type 2 diabetes, although the limited number and high heterogeneity of available studies suggests caution in drawing definitive conclusions. In patients with T2DM, studies performed in Asia showed a significant reduction in HbA1c, which was not observed in European studies. This geographic difference could have been determined by the differences in confinement measures (much stricter in Europe than in some Asian countries such as South Korea [30]), or by cultural differences possibly affecting the effect of lockdown on diet and physical activity; in studies performed in India, for example, lockdown appeared to have only a minor effect on physical activity [22, 23].

Some limitations of the present meta-analysis should be recognized. Centers performing the studies were often third-level clinics, which are not representative of all diabetes care facilities, because of a possible wider use of telemedicine, continuous glucose monitoring and more advanced treatments. Most of the studies performed in T1DM patients, furthermore, only enrolled patients which had performed at least 70% scans, thus excluding less compliant patients, who may be at higher risk of glucose deterioration. In addition, in our metanalysis, the mean age of the included patients with T2DM was low; accordingly, a survey has shown that patients contacted for telemedicine by a diabetes clinic during the pandemic were younger, with shorter disease duration and a lower prevalence of complications than the average pre-lockdown patients [31].

In conclusion, lockdown showed no significant detrimental effect on HbA1c in either T1DM or T2DM. Conversely, home confinement during the first pandemic wave led to a reduction in mean glucose and glucose variability in T1DM, although further studies are needed to better understand the high heterogeneity of results.

## Supplementary Information

Below is the link to the electronic supplementary material.

Supplementary file1 (DOCX 607 KB)

Supplementary file2 (DOCX 41 KB)
