# Supplementary material for: Glucose control in diabetes during home confinement for the first pandemic wave of COVID-19: a meta-analysis of observational studies
Source: Acta Diabetol. 2021 Jun 22;58(12):1603–11. doi: 10.1007/s00592-021-01754-2 (PMC8219181; doi:10.1007/s00592-021-01754-2)
Supplement: Supplementary file 1 — Supplementary file1 (DOCX 607 KB) [file 592_2021_1754_MOESM1_ESM.docx]

| PUBMED search string: ("sars cov 2"[MeSH Terms] OR "sars cov 2"[All Fields] OR "covid"[All Fields] OR "covid 19"[MeSH Terms] OR "covid 19"[All Fields]) AND ("diabetes mellitus"[MeSH Terms] OR ("diabetes"[All Fields] AND "mellitus"[All Fields]) OR "diabetes mellitus"[All Fields]) |
| --- |
| EMBASE search string: covid 19 AND diabetes AND mellitus AND [embase]/lim NOT ([embase]/lim AND [medline]/lim) |

Table 1S: Search String


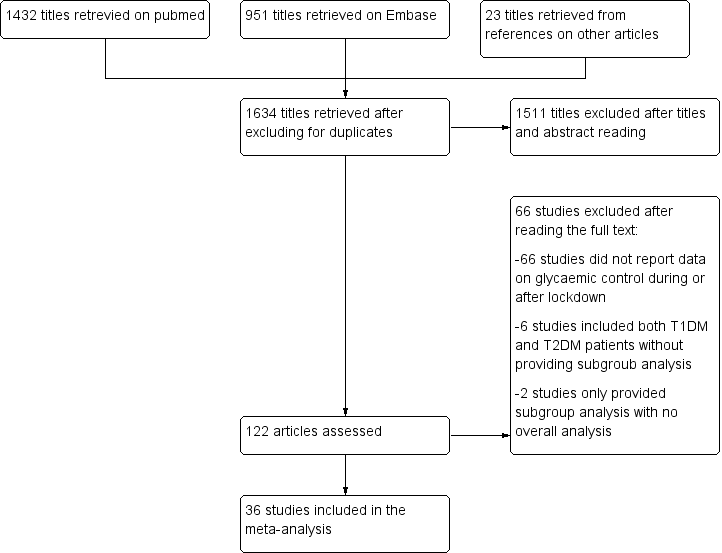


Fig. 1S: Trial Research Flow

| **Major Components** | **Judgment** |
| --- | --- |
| 1. Is the hypothesis/aim/objective of the study clearly stated? 2. Are the characteristics of the participants included in the study described? 3. Were the cases collected in more than one centre? 4. Are the eligibility criteria (i.e. inclusion and exclusion criteria) for entry into the study clearly stated? 5. Wereparticipantsrecruitedconsecutively? 6. Did participants enter the study at a similar point in the disease? 7. Was the intervention of interest clearly described? 8. Were additional interventions (co-interventions) reported in the study? 9. Are the outcome measures established a priori? 10. Were the relevant outcomes measured with appropriate objective and/or subjective methods? 11. Were the relevant outcomes measured before and after the intervention? 12. Were the statistical tests used to assess the relevant outcomes appropriate? 13. Was the length of follow-up reported? 14. Was the loss to follow-up reported? 15. Does the study provide estimates of the random variability in the data analysis of relevant outcomes? 16. Are the adverse events related with the intervention reported? 17. Are the conclusions of the study supported by results? 18. Are both competing interests and sources of support for the study reported? | 1. Yes, Unclear, No 2. Yes, Partially reported, No 3. Yes, Unclear, No 4. Yes, Partially reported, No 5. Yes, Unclear, No 6. Yes, Unclear, No 7. Yes, Partially reported, No 8. Yes, Unclear, No 9. Yes, Partially reported, No 10. Yes, Unclear, No 11. Yes, Unclear, No 12. Yes, Unclear, No 13. Yes, Unclear, No 14. Yes, Unclear, No 15. Yes, Unclear/partially reported, No 16. Yes, Partially reported, No 17. Yes, Partially reported, No 18. Yes, Partially reported, No |

Table 2S: the Carmen-Moga scale for risk of bias

| **Study name** | **CM1** | **CM2** | **CM3** | **CM4** | **CM5** | **CM6** | **CM7** | **CM8** | **CM9** | **CM10** | **CM11** | **CM12** | **CM13** | **CM14** | **CM15** | **CM16** | **CM17** | **CM18** |
| --- | --- | --- | --- | --- | --- | --- | --- | --- | --- | --- | --- | --- | --- | --- | --- | --- | --- | --- |
| Al Agha 2021 | ⚫ | ⚫ | ⚫ | ⚫ | ⚫ | 🔾 | ⚫ | ⚫ | ⚫ | ⚫ | ⚫ | ⚫ | 🔾 | ⚫ | 🔾 | 🔾 | ⚫ | ⚫ |
| Aragona 2020 | ⚫ | ⚫ | 🔾 | ⚫ | ⚫ | 🔾 | ⚫ | 🔾 | ⚫ | ⚫ | ⚫ | ⚫ | 🔾 | 🔾 | 🔾 | 🔾 | ⚫ | ⚫ |
| Barchetta 2020 | ⚫ | ⚫ | 🔾 | ⚫ | ⚫ | 🔾 | ⚫ | ⚫ | ⚫ | ⚫ | ⚫ | ⚫ | ⚫ | 🔾 | 🔾 | ⚫ | ⚫ | ⚫ |
| Barmpagianni, 2021 | ⚫ | ⚫ | ⚫ | ⚫ | ⚫ | 🔾 | ⚫ | ⚫ | ⚫ | ⚫ | ⚫ | ⚫ | 🔾 | ⚫ | 🔾 | 🔾 | ⚫ | ⚫ |
| Brener | ⚫ | ⚫ | ⚫ | ⚫ | ⚫ | 🔾 | ⚫ | ⚫ | ⚫ | ⚫ | ⚫ | ⚫ | 🔾 | ⚫ | 🔾 | 🔾 | ⚫ | ⚫ |
| Capaldo 2020 | ⚫ | ⓿ | 🔾 | ⚫ | ⚫ | 🔾 | ⚫ | 🔾 | ⚫ | ⚫ | ⚫ | ⚫ | 🔾 | 🔾 | 🔾 | 🔾 | ⚫ | ⚫ |
| Caruso | ⚫ | ⓿ | 🔾 | ⚫ | ⚫ | 🔾 | ⚫ | 🔾 | ⚫ | ⚫ | ⓿ | ⚫ | ⓿ | ⚫ | 🔾 | ⚫ | ⚫ | ⚫ |
| Ceconi | ⚫ | ⚫ | ⚫ | ⚫ | ⚫ | 🔾 | ⚫ | ⚫ | ⚫ | ⚫ | ⚫ | ⚫ | 🔾 | ⚫ | 🔾 | 🔾 | ⚫ | ⚫ |
| Di Dalmazi 2020 | ⚫ | ⚫ | ⚫ | ⚫ | ⚫ | 🔾 | ⚫ | ⚫ | ⚫ | ⚫ | ⚫ | ⚫ | 🔾 | ⚫ | 🔾 | 🔾 | ⚫ | ⚫ |
| Dover 2021 | ⚫ | ⚫ | ⚫ | ⚫ | ⚫ | 🔾 | ⚫ | ⚫ | ⚫ | ⚫ | ⚫ | ⚫ | 🔾 | 🔾 | 🔾 | 🔾 | ⚫ | ⚫ |
| Fernandez 2020 | ⚫ | ⓿ | ⚫ | ⚫ | ⚫ | 🔾 | ⚫ | 🔾 | ⚫ | ⚫ | ⚫ | ⚫ | 🔾 | 🔾 | 🔾 | 🔾 | ⚫ | ⚫ |
| Marigliano 2020 | ⚫ | ⚫ | 🔾 | ⚫ | ⚫ | 🔾 | ⚫ | 🔾 | ⚫ | ⚫ | ⚫ | ⚫ | ⚫ | ⚫ | ⚫ | 🔾 | ⚫ | ⚫ |
| Mesa 2020 | ⚫ | ⓿ | 🔾 | ⚫ | ⚫ | 🔾 | ⚫ | 🔾 | ⚫ | ⚫ | ⚫ | ⚫ | 🔾 | 🔾 | 🔾 | 🔾 | ⚫ | ⚫ |
| Moreno-Dominguez 2021 | ⚫ | ⚫ | 🔾 | ⚫ | ⚫ | 🔾 | ⚫ | 🔾 | ⚫ | ⚫ | ⚫ | ⚫ | 🔾 | 🔾 | 🔾 | 🔾 | ⚫ | ⚫ |
| Pla 2020 | ⚫ | ⓿ | 🔾 | ⚫ | ⚫ | 🔾 | ⚫ | 🔾 | ⚫ | ⚫ | ⚫ | ⚫ | 🔾 | 🔾 | 🔾 | 🔾 | ⚫ | ⚫ |
| Predieri 2020 | ⚫ | ⓿ | 🔾 | ⚫ | ⚫ | 🔾 | ⚫ | 🔾 | ⚫ | ⚫ | ⚫ | ⚫ | 🔾 | 🔾 | 🔾 | 🔾 | ⚫ | ⚫ |
| Shah2020 | ⚫ | ⚫ | ⚫ | ⚫ | ⚫ | 🔾 | ⚫ | ⚫ | ⚫ | ⚫ | ⚫ | ⚫ | 🔾 | ⚫ | 🔾 | 🔾 | ⚫ | ⚫ |
| Verma 2020 | ⚫ | ⓿ | 🔾 | ⓿ | ⚫ | 🔾 | ⚫ | 🔾 | ⚫ | ⚫ | ⚫ | ⚫ | 🔾 | 🔾 | 🔾 | 🔾 | ⚫ | ⚫ |
| Vinals 2020 | ⚫ | ⚫ | 🔾 | ⚫ | ⚫ | 🔾 | ⚫ | ⚫ | ⚫ | ⚫ | ⚫ | ⚫ | 🔾 | 🔾 | 🔾 | ⚫ | ⚫ | ⚫ |
| Cotovad-Bellas 2021 | ⚫ | ⚫ | 🔾 | ⚫ | ⚫ | 🔾 | ⚫ | ⚫ | ⚫ | ⚫ | ⚫ | ⚫ | ⚫ | ⚫ | ⚫ | 🔾 | ⚫ | ⚫ |
| Prabhu Navis 2020 | ⚫ | ⚫ | 🔾 | ⚫ | ⚫ | 🔾 | ⚫ | 🔾 | ⚫ | ⚫ | ⚫ | ⚫ | ⚫ | ⚫ | 🔾 | ⚫ | ⚫ | ⚫ |
| Christoforidis 2020 | ⚫ | ⚫ | 🔾 | ⚫ | ⚫ | 🔾 | ⚫ | 🔾 | ⚫ | ⚫ | ⚫ | ⚫ | ⚫ | ⚫ | 🔾 | ⚫ | ⚫ | ⚫ |
| Dovc 2020 | ⚫ | ⚫ | 🔾 | ⚫ | ⚫ | 🔾 | ⚫ | 🔾 | ⚫ | ⚫ | ⚫ | ⚫ | ⚫ | ⚫ | 🔾 | ⚫ | ⚫ | ⚫ |
| Longo 2020 | ⚫ | ⚫ | 🔾 | ⚫ | ⚫ | 🔾 | ⚫ | 🔾 | ⚫ | ⚫ | ⚫ | ⚫ | ⚫ | ⚫ | 🔾 | ⚫ | ⚫ | ⚫ |
| Schiaffini 2020 | ⚫ | ⚫ | 🔾 | ⚫ | ⚫ | 🔾 | ⚫ | 🔾 | ⚫ | ⚫ | ⚫ | ⚫ | ⚫ | ⚫ | 🔾 | ⚫ | ⚫ | ⚫ |
| Anjana 2020 | ⚫ | ⚫ | 🔾 | ⚫ | ⚫ | 🔾 | ⚫ | 🔾 | ⚫ | ⚫ | ⚫ | ⚫ | 🔾 | 🔾 | 🔾 | 🔾 | ⚫ | ⚫ |
| Biancalana 2020 | ⚫ | ⚫ | 🔾 | ⚫ | ⚫ | 🔾 | ⚫ | 🔾 | ⚫ | ⚫ | ⚫ | ⚫ | 🔾 | 🔾 | 🔾 | 🔾 | ⚫ | ⚫ |
| Karatas 2021 | ⚫ | ⚫ | 🔾 | ⚫ | ⚫ | 🔾 | ⚫ | 🔾 | ⚫ | ⚫ | ⚫ | ⚫ | 🔾 | 🔾 | 🔾 | 🔾 | ⚫ | ⚫ |
| Onmez 2020 | ⚫ | ⚫ | 🔾 | ⚫ | ⚫ | 🔾 | ⚫ | ⚫ | ⚫ | ⚫ | ⚫ | ⚫ | 🔾 | 🔾 | 🔾 | 🔾 | ⚫ | ⚫ |
| Sung-Don Park 2021 | ⚫ | ⚫ | 🔾 | ⚫ | ⚫ | 🔾 | ⚫ | ⚫ | ⚫ | ⚫ | ⚫ | ⚫ | 🔾 | ⚫ | 🔾 | 🔾 | ⚫ | ⚫ |
| Tourkmani 2020 | ⚫ | ⚫ | 🔾 | ⚫ | ⚫ | 🔾 | ⚫ | ⚫ | ⚫ | ⚫ | ⚫ | ⚫ | 🔾 | ⚫ | 🔾 | 🔾 | ⚫ | ⚫ |
| Sankar 2020 | ⚫ | ⚫ | 🔾 | ⚫ | ⚫ | 🔾 | ⚫ | ⚫ | ⚫ | ⚫ | ⚫ | ⚫ | 🔾 | 🔾 | 🔾 | 🔾 | ⚫ | ⚫ |
| Munekawa 2020 | ⚫ | ⚫ | 🔾 | ⚫ | ⚫ | 🔾 | ⚫ | ⚫ | ⚫ | ⚫ | ⚫ | ⚫ | ⚫ | 🔾 | ⓿ | 🔾 | ⚫ | ⚫ |

Table 3S: Risk of bias assessed according to the Carmen-Moga scale

Figure 3S: HbA1c in T1DM before and after lockdown: subgroup analysis for proportion of patients on SMBG

Figure 2S: Hba1c in T1DM before and after lockdown: subgroup analysis for age

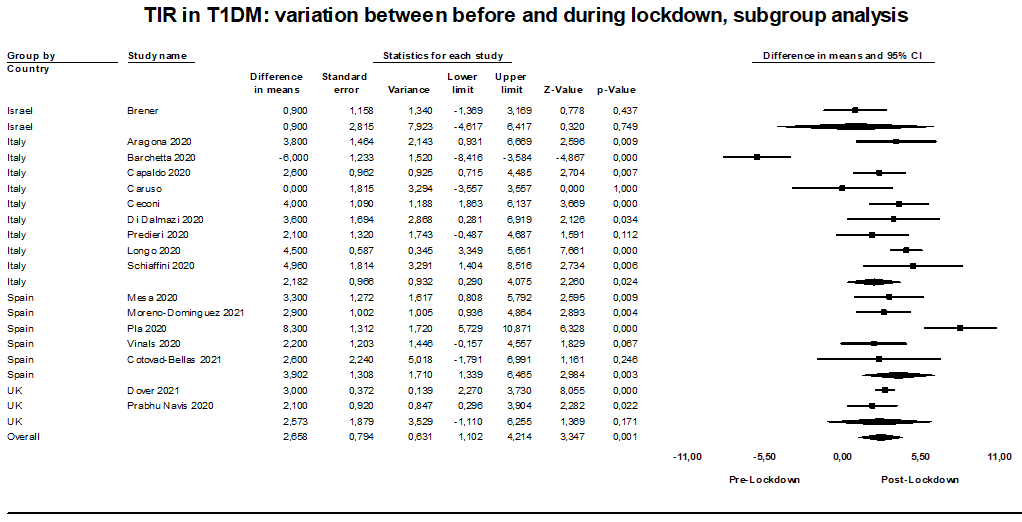


Figure 5S: TIR in T1DM before and during lockdown: subgroup analysis for different Countries

Figure 4S: funnel plot for TIR pre and during lockdown

| **Moderators** | **R** | **Lower limit** | **Upper limit** | **p-value** |
| --- | --- | --- | --- | --- |
| **% on CSII** | 0.006 | -0.007 | 0.02 | 0.36 |
| **% on FGM** | 0.002 | -0.03 | 0.15 | 0.88 |
| **% on CGM** | -0.002 | -0.03 | 0.28 | 0.88 |
| **% on CGM or FGM** | 0.69 | -4.34 | 5.73 | 0.78 |
| **Duration of DM** | 0.058 | -0.14 | 0.26 | 0.56 |
| **Baseline TIR** | -0.07 | -0.138 | -0.005 | 0.036 |
| **% of males** | -0.09 | -0.16 | -0.026 | 0.006 |
| **Mean age** | -0.016 | -0.06 | 0.03 | 0.50 |

Table 4S: Meta-regression analysis for TIR before and during lockdown

Figure 6S: TIR in T1DM before and during lockdown: meta regression analysis for proportion of males

Figure 7S: TIR in T1DM, before and during lockdown


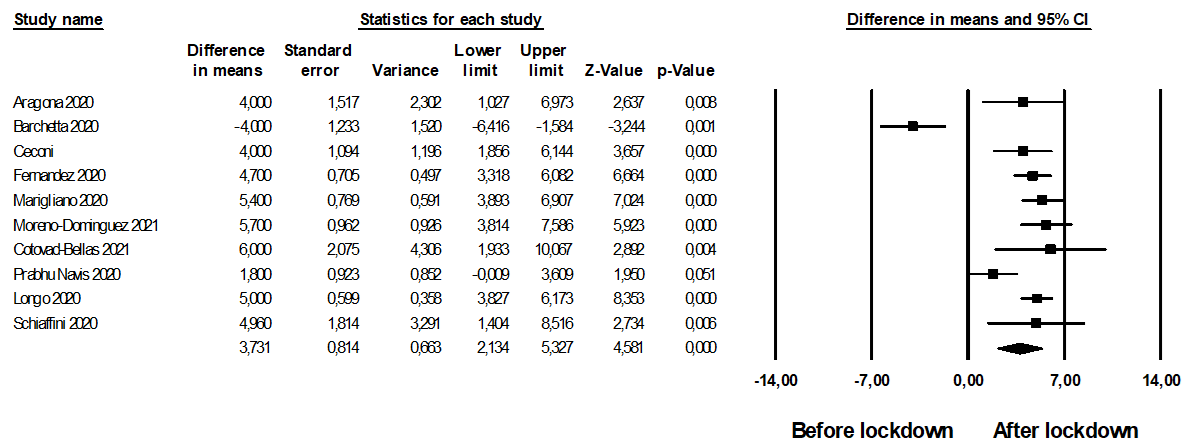


Figure 8S: TIR in T1DM before and after lockdown


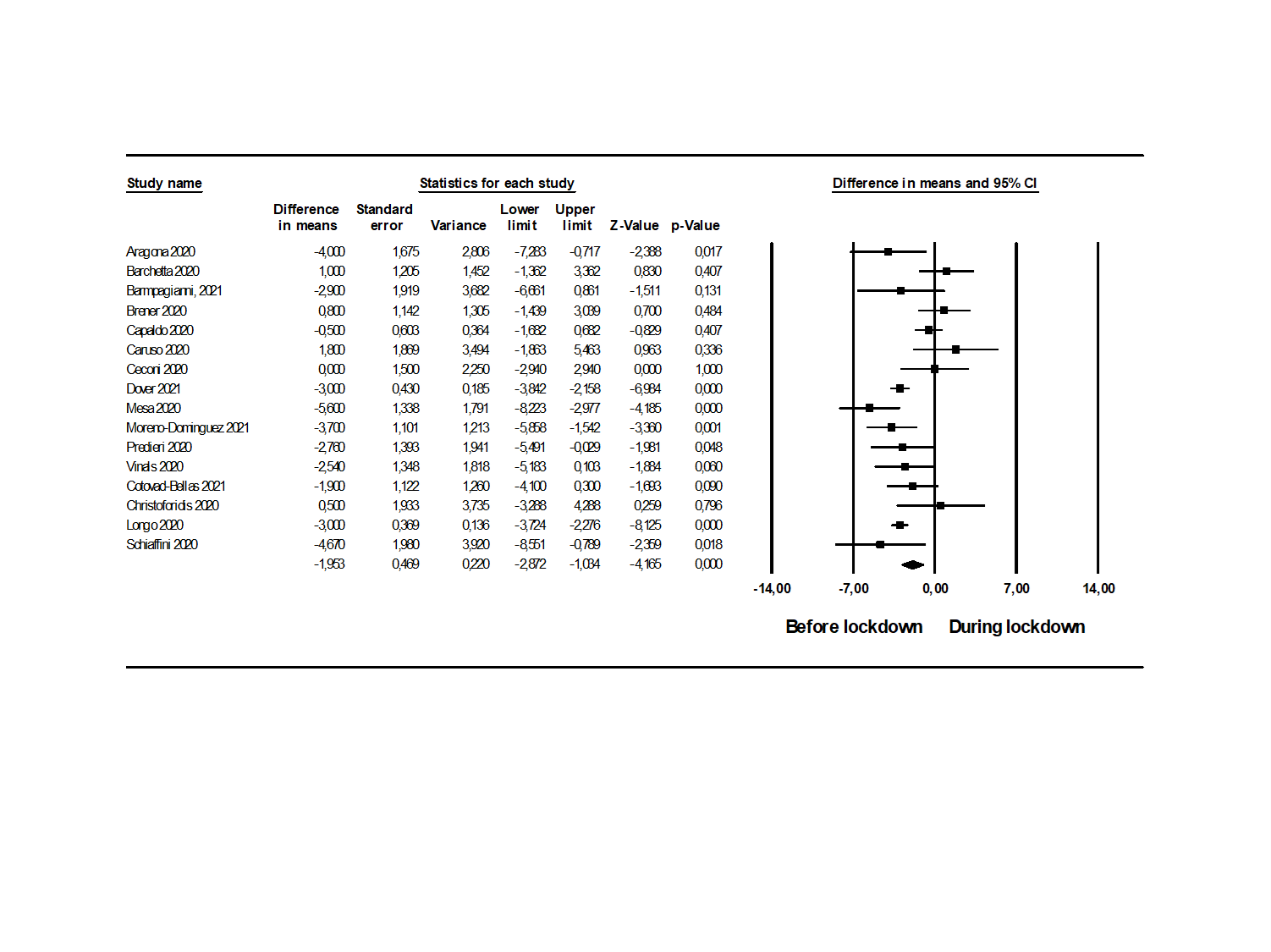


Figure 9S: TAR in T1DM before and during lockdown


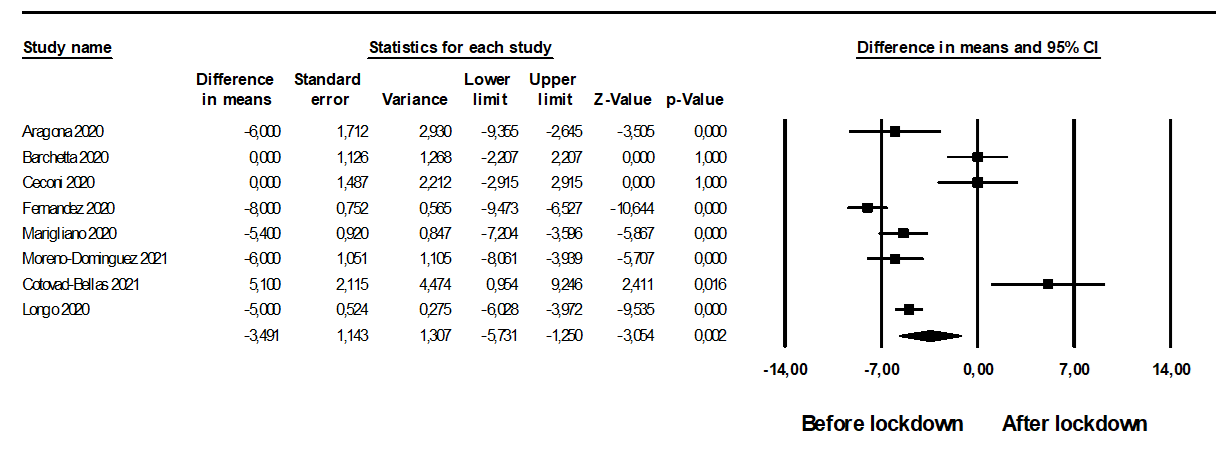


Figure 10S: TAR in T1DM before and after lockdown

Figure 11S: TBR in T1DM before and during lockdown

Figure 12S: TBR in T1DM before and after lockdown

Figure 14S: mean glucose in T1DM before and after lockdown

Figure 13S: mean glucose in T1DM before and during lockdown

Figure 15S: Glucose coefficient of variability in T1DM before and during lockdown

Figure 16S: Glucose coefficient of variability in T1DM before and after lockdown

Figure 17S: HbA1c in T2DM before and after lockdown: subgroup analysis for basaline HbA1c

Figure 19S: HbA1c in T2DM before and after lockdown: subgroup analysis for Age

Figure 18S: HbA1c in T2DM before and after lockdown: subgroup analysis for continent in which the study was performed

Figure 16S: Glucose coefficient of variability in T1DM before and after lockdown
