# Supplementary material for: Glucose control in diabetes during home confinement for the first pandemic wave of COVID-19: a meta-analysis of observational studies
Source: Acta Diabetol. 2021 Jun 22;58(12):1603–11. doi: 10.1007/s00592-021-01754-2 (PMC8219181; doi:10.1007/s00592-021-01754-2)
Supplement: Supplementary file 2 — Supplementary file2 (DOCX 41 KB) [file 592_2021_1754_MOESM2_ESM.docx]

| **Section/topic** | | | | | **#** | **Checklist item** | **Reported on page #** |
| --- | --- | --- | --- | --- | --- | --- | --- |
| **TITLE** | | | | | | |  |
| Title | | | | | 1 | Identify the report as a systematic review, meta-analysis, or both.  *“Glucose control in diabetes during home confinement for the first pandemic wave of COVID-19: a meta-analysis of observational studies”* | 1 |
| **ABSTRACT** | | | | | | |  |
| Structured summary | | | 2 | Provide a structured summary including, as applicable: background; objectives; data sources; study eligibility criteria, participants, and interventions; study appraisal and synthesis methods; results; limitations; conclusions and implications of key findings; systematic review registration number.  *“Aim: To assess the effect on glycemic control of confinement due to lockdown measures, during COVID-19 pandemic, in people with type 1 (T1DM) and type 2 (T2DM) diabetes. Methods: meta-analysis of observational studies reporting measures of glucose control and variability before and during and/or after periods of confinement caused by COVID-19 in 2020 and/or 2021.  Results: We included 27 studies on T1DM. No significant change in Hba1c was observed after lockdown (WMD -1.474 [-3.26; 0.31] mmol/mol, I2= 93.9). TIR significantly increased during and after lockdown (WMD: 2.73 [1.47; 4.23]%, I2= 81% and 3.73 [1.13; 5.33]%, I2= 85%, respectively). We retrieved nine studies on T2DM patients. No significant variation in HbA1c was detected (WMD -1.257 [-3.91; 1.39] mmol/mol, I2= 98,3%). HbA1c had a more favourable trend in studies performed in Asia than in Europe (p=0.022 between groups). Conclusion: lockdown showed no significant detrimental effect on HbA1c in either T1DM and T2DM. Conversely, home confinement led to a reduction of mean glucose and glucose variability in T1DM, although with a high heterogeneity of results.”.* | | | 1 |
| **INTRODUCTION** | | | | | | |  |
| Rationale | 3 | Describe the rationale for the review in the context of what is already known. *COVID-19 pandemic forced most Countries to adopt confinement measures to prevent the spreading of the disease. Those measures, although different across countries, all led to some extent to a reduction in physical activity, with the shutdown of gyms in many countries; moreover, the commitment to stay at home modified daily routine, increasing the time available for cooking and eating. All those changes in daily routine may have altered glycemic control in people with diabetes mellitus. The outbreak of COVID-19 during the spring of 2020 and the consequent lockdown in many countries also reduced the access to diabetes specialist care, metabolic monitoring through laboratory determination, and visits and exams for screening of diabetic complications. The reduced availability of medical care, associated with insufficient patient self-management , was a possible determinant of the observed increase in incidence and severity of diabetic complications, such as foot ulcers . In order to maintain basic care, tele monitoring was implemented in many countries. Telematic interactions were also used to help patients in developing coping strategies for managing home confinement] and maintaining physical activity. Physical activity is crucial in diabetes mellitus management, especially during a pandemic, as it enhances immune response to viral infections; therefore, many efforts have been performed to help patients in finding strategies to maintain it during lockdown. The success of strategies implemented for the care of diabetes during prolonged lockdown for COVID-19 epidemic waves was assessed in several observational studies, providing discordant results.* | | | | | 1 |
| Objectives | 4 | Provide an explicit statement of questions being addressed with reference to participants, interventions, comparisons, outcomes, and study design (PICOS).  *This meta-analysis is aimed at collecting all evidence on the effect on glycemic control of confinement due to lockdown measures, and the consequent adaptation of care, during the first wave of COVID-19 pandemic, in patients with type 1 and type 2 diabetes.* | | | | | 2 |
| **METHODS** | | | | | | |  |
| Protocol and registration | | | | | 5 | Indicate if a review protocol exists, if and where it can be accessed (e.g., Web address), and, if available, provide registration information including registration number.  *Review Protocol has been submitted for registration to the PROSPERO website CRD42021234360https://www.crd.york.ac.uk/PROSPERO/* | 2 |
| Eligibility criteria | | | | | 6 | Specify study characteristics (e.g., PICOS, length of follow-up) and report characteristics (e.g., years considered, Observational studies written in English language and performed on humans, enrolling patients with type 1 or type 2 diabetes, and reporting measures of glucose control and variability before and during and/or after periods of confinement caused by COVID-19 in 2021 and/or 2021 were included. | 2 |
| Information sources | | | | | 7 | Describe all information sources (e.g., databases with dates of coverage, contact with study authors to identify additional studies) in the search and date last searched.  *For all published studies, results reported in published papers and supplementary materials were used as the source of information.* | 3 |
| Search | | | | | 8 | Present full electronic search strategy for at least one database, including any limits used, such that it could be repeated.  *Searches were performed in PubMed and Embase (“COVID-19” AND “diabetes mellitus”, no limits a) up to March, 10th, 2021 (see the complete search strings in Supplementary Table 1). Further studies were searched among references from papers.* | 3, tab.1S of suppl. materials |
| Study selection | | | | | 9 | State the process for selecting studies (i.e., screening, eligibility, included in systematic review, and, if applicable, included in the meta-analysis).   *Titles and abstracts were screened independently by two auhtors. If one or more inclusion criteria was present, the whole article was read, in order to assess if all the inclusion criteria were present* | 3 |
| Data collection process | | | | | 10 | Describe method of data extraction from reports (e.g., piloted forms, independently, in duplicate) and any processes for obtaining and confirming data from investigators. *For all published trials, results reported in published papers and supplementary materials were used as the primary source of information. Study selection, data retrieval, and study quality assessment were performed independently by two investigators (C.D.P. and A.S.), and conflicts resolved by a third investigator (M.M.)..* | 3 |
| Data items | | | | | 11 | List and define all variables for which data were sought (e.g., PICOS, funding sources) and any assumptions and simplifications made. *number of included patients, duration of diabetes, mean age, proportion of proportion of male patients, patients using flash glucose monitoring (FGM), continuous glucose monitoring (CGM), or self monitoring of blood glucose (SMBG); proportion of patients in continuous subcutaneous insulin infusion (CSII) multiple daily insulin injections (MDI), basal insulin only, sodium glucose transporter 2 inhibitors (SGLT2-i), dipeptiydil-4 inhibitors (DPP-4i), pioglitazone, metformin, sulphonylureas (SU); study duration, country of origin, duration of Lockdown, use of teleconsulting, values of HbA1c before and after lockdown, TIR,TAR, ,TBR, proportion of time in which glucose is below 70 mg/dl), Coefficient of variability (CV), use of telemedicine, any variation in physical activity, diet, stress; all data were collected, before, during o*r after the lockdown. | 3 |
| Risk of bias in individual studies | | | | | 12 | Describe methods used for assessing risk of bias of individual studies (including specification of whether this was done at the study or outcome level), and how this information is to be used in any data synthesis.  *The quality of the studies was assessed at study level, using the scale developed by Carmen-Moga and collegues.* | 3, *table 2 of Suppl. materials* |
| Summary measures | | | | | 13 | State the principal summary measures (e.g., risk ratio, difference in means).  *Between-group difference-in means with 95% confidence Interval were calculated.* | 4 |
| Synthesis of results | | | | | 14 | Describe the methods of handling data and combining results of studies, if done, including measures of consistency (e.g., I^2^) for each meta-analysis.  *Heterogeneity was assessed by using I2 statistics. A random-effects model was applied as the primary analysis.* | 4 |

Page 1 of 2

| **Section/topic** | **#** | **Checklist item** | **Reported on page #** |
| --- | --- | --- | --- |
| Risk of bias across studies | 15 | Specify any assessment of risk of bias that may affect the cumulative evidence (e.g., publication bias, selective reporting within studies). *Begg’s and Mandzumkar test were used to detect publication bias, with reference to all principal endpoints; funnel plots were used when more than 10 studies were available for the specific outcome-* | 4 |
| Additional analyses | 16 | Describe methods of additional analyses (e.g., sensitivity or subgroup analyses, meta-regression), if done, indicating which were pre-specified. Subgroup analyses were performed, based on Country, age group (children and adolescent <18, adult >18 years), type of monitoring (FGM, CGM, SMBG), insulin treatment (multiple injections, continuous subcutaneous infusion, hybrid closed loop systems), structured tele-monitoring (yes/no). | 4 |
| **RESULTS** | | |  |
| Study selection | 17 | Give numbers of studies screened, assessed for eligibility, and included in the review, with reasons for exclusions at each stage, ideally with a flow diagram.  *Out of 1634 results, 122 studies were selected on the basis of the titles and abstracts. Of those, 79 did not report data on glycemic control during or after lockdown measures; 6 included both T1DM and T2DM patients without providing subgroup analysis; two studies reported subgroup analyses with no overall analysis. Thirty-six studies reported glycemic control before and during or after the pandemic restrictions and were therefore included in the meta-analysis (trial research flow at Fig.1S). Of those, 9 were performed in type 2 diabetes, whereas 27 were performed in type 1 diabetes. Characteristics of the included studies were reported in table 1..* | 4, tab.1, *Fig.1S* |
| Study characteristics | 18 | For each study, present characteristics for which data were extracted (e.g., study size, PICOS, follow-up period) and provide the citations.  *The main characteristics of included trials are reported in Table 1. Out of 35 studies, 27 excluded patients with kidney disease, 18 studies excluded patients with previous CVD disease. One study reported two different comparisons between low carbohydrate and balanced diets: results of each comparison is therefore reported separately.* | 4,  table 1 |
| Risk of bias within studies | 19 | Present data on risk of bias of each study and, if available, any outcome level assessment (see item 12).  *Risk of bias is reported in Fig. 2S and 3S of Supplementary materials* | 4, Fig. 2S-3S |
| Results of individual studies | 20 | For all outcomes considered (benefits or harms), present, for each study: (a) simple summary data for each intervention group (b) effect estimates and confidence intervals, ideally with a forest plot.  *Forest plots for main outcomes are reported in fig.1, fig.2 Forest plots for secondary outcomes are reported in supplementary materials, fig. 5-72S and tab*  *Out of 1634 results, 122 studies were selected on the basis of the titles and abstracts. Of those, 79 did not report data on glycemic control during or after lockdown measures; 6 included both T1DM and T2DM patients without providing subgroup analysis[17]; two studies reported subgroup analyses with no overall analysis[22]. Thirty-six studies reported glycemic control before and during or after the pandemic restrictions and were therefore included in the meta-analysis (Fig.1S). Of those, 9 were performed in type 2 diabetes, whereas 27 were performed in type 1 diabetes. Characteristics of the included studies were reported in table 1.*  *HbA1c. Only 9 studies, enrolling 1174 patients, reported HbA1c before and after lockdown in patients with type 1 diabetes.*  *Nine studies reporting HbA1c before and after lockdown were available in T2DM patients, including 9,591 subjects with a median age of 60.5 years; five studies were performed in Asia (India, South Korea, Japan, Saudi Arabia), whereas four were performed in Europe (Turkey, Italy, Greece).* | 3-5  Fig.1-2 Tab.2 Fig 7S-16SS. |
| Synthesis of results | 21 | Present results of each meta-analysis done, including confidence intervals and measures of consistency.  Trial characteristics  *Type 1 diabetes: No significant change in Hba1c was observed after lockdown (WMD -1.474 [-3.26; 0.31] mmol/mol; Fig.1 panel A), with relevant heterogeneity (I2= 93.9). No publication bias was detected (Kendall’s tau:16 p= 0.1). Time in range: Nineteen studies, enrolling 1,985 patients, and 10 studies, enrolling 1,123 patients, reported information on TIR during and after lockdown, respectively. TIR significantly increased during lockdown (WMD: 2.73 [1.47; 4.23]%; fig.2), with high heterogeneity (I2= 81%) and no detectable publication bias (Kendall’s tau: 0.1, p= 0.59; fig. 2S, for funnel plot). Time above range: TAR during lockdown and after lockdown was reported in 14 and 9 studies, respectively. TAR was significantly lower both during (WMD: -1.953 [-2.87;-1.03] I2= 70, Kendall’s tau:7. p= 0.7 (Fig.9S) and after lockdown (WMD: -3.49 [-0.57; -1.25] I2=90 Kendall’s tau:10. p= 0.21); (Fig.10S).*  *Time below range: Seventeen studies on type 1 diabetes estimated time below range (TBR) before and during lockdown, whereas nine studies reported TBR before and after lockdown: TBR did not change significantly during (WMD: 0.13 [-0.18; 0.43]; I2: 81% and Kendall’s tau: 0.1, p= 0.59; Fig.11S) or after lockdown (WMD: 0.29 [-0.28; 0.86); I2= 94 and Kendall’s tau:12. p= 0.21; Fig.12S).*  *Mean glucose. In the 14 studies with available data, mean glucose during lockdown was significantly lower than before lockdown (WMD -2.795 [-4.816; -0.774] (Fig.13S), with high heterogeneity (I2= 91) and no evidence of publication bias (Kendall’s tau: -11.0 p= 0.54). In addition, mean glucose was significantly lower after lockdown (WMD -5.29 [-8.055; -2.53] with high heterogeneity (I2= 87.989) and no evidence of publication bias. (Kendall’s tau: -9 p= 0.17; fig.14S).*  *Glucose coefficient of variation. Glucose CV during (n=16 studies) and after (n=9 studies) lockdown was significantly reduced (WMD: -0.97 [-1.48; -0.47]; Fig.15S and -1.33 [-2.11; -0.56]; Fig.16S, respectively), with no evidence of publication bias (Kendall’s tau=-29.0; p=0.27) and high heterogeneity (I2= 79).*  *Patients’ reported behaviours: Thirteen studies enrolling patients with T1DM reported data on patients’ behaviours. The heterogeneity of instruments used for the assessment of patients’ behaviour prevented a formal meta-analysis (tab 4). A reduction in physical activity was reported by 8-70% of patients, whereas those reporting an increase in food intake were 17-46%; moderate-to high stress was found in 20-52% of patients.*  *Type 2 diabetes: No significant variation in HbA1c was detected (WMD -1.257 [-3.91; 1.39] mmol/mol; fig. 1, panel B), with high heterogeneity (I2= 98,3%). No significant publication bias was detected (Kendall’s tau=-1, p=0.88). A subgroups analysis revealed a significant difference between studies with mean baseline HbA1c below or above 64 mmol/mol (p=0.045 between groups), with those with higher baseline HbA1c showing a greater reduction (Fig.17S). A further subgroup analysis showed that HbA1c had a more favourable trend in studies performed in Asia than in Europe (p=0.022 between groups) (Fig.18S). No difference was found between age groups (p=0.22 between studies with a mean age higher or lower than 60 years) (Fig.19S).*  *Two studies on people with T2DM [23] [24] both performed in India, reported a modest reduction (20-24% of participants) in physical activity with no significant variation in food intake. On the other hand, two studies performed in Turkey and Japan [25] [26], reported a frequent (54-70% of participants) reduction in physical activity and an increase in food intake (20-55% of participants). All the three studies reporting data on stress found a moderate increase in perceived stress and anxiety (Table 4).* | #3-5  Fig.1-2 Tab.2 Fig 7S-16SS. |
| Risk of bias across studies | 22 | Present results of any assessment of risk of bias across studies (see Item 15). No significant publication bias was found for any outcome (See results section). Risk of bias assessed through the Carmen Moga Scale was | #3-5 Tab.2S,3S, Fig. 4S |
| Additional analysis | 23 | Give results of additional analyses, if done (e.g., sensitivity or subgroup analyses, meta-regression [see Item 16]).  *A subgroup analysis showed that studies with more than 50% of enrolled patients on continuous glucose monitoring showed a significant decrease in HbA1c (WMD -3.00 [-4.84; -1.16] mmol/mol), whereas those enrolling more than 50% patients on SMBG showed no significant variation in HbA1c (p=0.003 for difference between groups; fig. 5S). Studies performed in Europe showed a significant reduction in HbA1c (WMD -3.053 [-3.9; -2.2] mmol/mol), whereas those performed in Asia did not (WMD; 2.36 [-7.50; 12.25] mmol/mol; p<0.0001 for difference between groups). No significant difference was found in subgroup analyses based on age (fig. 4S).*  *TIR: Subgroups analyses revealed no difference between studies performed in different countries or in different age groups (Fig. 7S). Meta-regression analysis showed no correlation of TIR variation with its baseline value, or with the proportion of subjects on CSII or MDI (Tab. 4S). Conversely, an inverse correlation was detected between variation in TIR and proportion of men among enrolled subjects (Tab 4S, Fig. 3S). TIR was significantly higher after lockdown (WMD 3.73 [1.13; 5.33] %; Fig. 8S) with high heterogeneity (I2= 85%). No significant publication bias was detected (Kendall’s tau: -7. p= 0.48).* | 3-5 Fig.2S,3S5S,6S, tab 4S,Fig.17S-19S |
| **DISCUSSION** | | |  |
| Summary of evidence | 24 | Summarize the main findings including the strength of evidence for each main outcome; consider their relevance to key groups (e.g., healthcare providers, users, and policy makers). Most studies on the glycemic effects of lockdown were performed in T1DM, usually in patients using either FGM or CGM. The assessment of variations in intersitial glucose can be can be performed in a shorter time than that required for exploring modifications in HbA1c. For this reason, we already have a substantial body of evidence on the effects of lockdown during the first pandemic wave in T1DM, but not in T2DM.  In T1DM, an improvement in glycemic control during lockdown was observed, together with a reduction of glucose variability. These results were obtained despite an observed reduction in physical activity and dietary compliance during lockdown, determined by the increased time spent at home; in addition, access to care was impaired during lockdown [27], and surveys on perceived glucose control revealed a high perceived difficulty in dealing with COVID-19 restrictions in people with T1DM [28].  The interpretation of results on glucose control in T1DM is problematic because of their high heterogeneity. The exploration of moderators of lockdown effect, using either subgroup analyses of trials or meta-regression, provides some further insight. Lockdown seems to have produced a greater beneficial effect on females than in males. This is consistent with a Chinese study in which males with T1DM had poorer glycemic control than females during COVID-19 lockdown[29]; previous findings showed that females with DM, when compared to males, elicited more frequently behaviors aimed at disease prevention, health promotion, and symptom recognition [30], which could have been of help in coping with confinement. An increase in glucose monitoring and an improvement in insulin titration during remote working or remote schooling may explain these improvement, as suggested by a study showing an improvement in glucose control only in patients working at home[22]; unfortunately, the information on glucose monitoring and on the proportion of patients on home schooling or working was insufficient to add these variables as moderators. Notably, in studies enrolling a majority of patients with T1DM with interstitial glucose monitoring systems, HbA1c was significantly reduced, suggesting that FGM or CGM could have been a relevant support during confinement. The difference in effects of lockdown between Asian and European studies could have been determined by the different proportion of patients on FGM/CGM (substantially higher in European studies), or to differences in lockdown measures (usually stricter in European countries).  No significant beneficial or detrimental effect of lockdown on glucose control was found in Type 2 diabetes, although the limited number and high heterogeneity of available studies suggests caution in drawing definitive conclusions. In patients with T2DM, studies performed in Asia showed a significant reduction in HbA1c, which was not observed in European studies. This geographic difference could have been determined by the differences in confinement measures (much stricter in Europe than in some Asian countries such as South Korea [31]), or by cultural differences possibly affecting the effect of lockdown on diet and physical activity; in studies performed in India, for example, lockdown appeared to have only a minor effect on physical activity | 5 |
| Limitations | 25 | Discuss limitations at study and outcome level (e.g., risk of bias), and at review-level (e.g., incomplete retrieval of identified research, reporting bias). *Some limitations of the present meta-analysis should be recognized. Centers performing the studies were often third-level clinics, which are not representative of all diabetes care facilities, because of a possible wider use of telemedicine, continuous glucose monitoring and more advanced treatments. Most of the studies performed in T1DM patients, furthermore, only enrolled patients which had performed at least 70% scans, thus excluding less compliant patients, who may be at higher risk of glucose deterioration. In addition, in our metanalysis, the mean age of the included patients with T2DM was low; accordingly, a survey has shown that patients contacted for telemedicine by a diabetes clinic during the pandemic were younger, with shorter disease duration and a lower prevalence of complications than the average pre-lockdown patients* | 6 |
| Conclusions | 26 | Provide a general interpretation of the results in the context of other evidence, and implications for future research. *In conclusion, lockdown showed no significant detrimental effect on HbA1c in either T1DM and T2DM. Conversely, home confinement during the first pandemic wave led to a reduction of mean glucose an glucose variability in T1DM, although further studies are needed to better understand the high heterogeneity of results.* | 6 |
| **FUNDING** | | |  |
| Funding | 27 | Describe sources of funding for the systematic review and other support (e.g., supply of data); role of funders for the systematic review. *This research was performed as a part of the institutional activity of the unit, with no specific funding* | #11 |

*From:*  Moher D, Liberati A, Tetzlaff J, Altman DG, The PRISMA Group (2009). Preferred Reporting Items for Systematic Reviews and Meta-Analyses: The PRISMA Statement. PLoS Med 6(7): e1000097. doi:10.1371/journal.pmed1000097

For more information, visit: **www.prisma-statement.org**. Page 2 of 2
